# GERDA: The German Election Database

**DOI:** 10.1038/s41597-025-04811-5

**Published:** 2025-04-14

**Authors:** Vincent Heddesheimer, Hanno Hilbig, Florian Sichart, Andreas Wiedemann

**Affiliations:** 1 Princeton University, Department of Politics, Princeton, NJ 08544 USA; 2 University of California, Davis, Department of Political Science, Davis, CA 95616 USA

**Keywords:** Politics, Social sciences

## Abstract

Elections are the key mechanism through which voters hold elected officials accountable. The partisan composition of local, state, and federal governments, in turn, shapes policy choices and public goods provision. Yet studying representation, government responsiveness, and partisan politics across multiple levels of government—especially at the local level—has been difficult due to inconsistently reported, incomplete, or insufficiently harmonized election data at small geographic scales. This paper introduces GERDA (https://www.german-elections.com/), a panel dataset of local, state, and federal election results in Germany at the municipality level spanning the past three decades. GERDA includes turnout and vote shares for all major parties and resolves challenges arising from municipal boundary changes and joint mail-in voting districts, yielding a consistent panel of municipalities in their 2021 boundaries. We also provide municipal and county boundary shapefiles to facilitate spatial analyses. Our dataset enables new research on partisan politics, policy responsiveness, and political representation at fine-grained geographic scales and over time.

## Background & Summary

In democracies, elections are the key link between voter preferences and government policy. Voters hold parties and politicians accountable through elections. Which party governs shapes which societal groups are represented in government and affects people’s daily lives through policy choices and public goods and services provision. Studying questions of representation and policy responsiveness is demanding on the data: first, these questions require election data for the level of government responsible for specific policy choices. For example, in many countries, policy choices about housing, police, and basic public goods provisions are delegated to the local level, while macro-economic or foreign policy choices are decided at the national/federal level. Local politics in particular has received increasing attention among social science researchers. Long understudied, researchers now recognize that local politics is a crucial part of the political system in democratic countries; voters often care deeply about outcomes produced by local governments^1^. Second, these questions often require data at small scales, such as municipalities, to understand the political responses of elected officials at different levels of government to local economic shocks. Third, these questions require panel data to dynamically study changes over time.

However, studying government responsiveness and representation across different levels of government and over time is notoriously difficult: data is incomplete, difficult to collect, or subject to challenges such as inconsistent reporting formats, varying electoral rules across states, and mail-in voting districts. Analyses over time are further hampered by administrative boundary changes. Even when local data is available, it is often not centrally compiled and must be individually collected from various sources, often as PDF documents^2^.

In this paper, we address these challenges by providing a new database of electoral results in Germany, Europe’s largest democracy. In part due to the country’s federal structure, no single source for municipal legislative election data exists. We collect municipal, state, and federal election results – described in detail below – and create a dataset that includes turnout and party vote shares for municipal, state, and federal elections at the level of the municipality, the smallest administrative unit in the country. We provide data for municipal council elections (Stadtrat/Gemeinderat) and federal elections since reunification (1990) and for state elections since the mid-2000s. In total, we present results for 10,789 municipalities, covering a total of 26 municipal election years (*N* = 64, 666), 12 state election years (*N* = 26, 899), and 9 federal election years (*N* = 96, 759). To our knowledge, this dataset constitutes the most complete data on municipal, state, and federal election results in Germany and provides harmonized data for all municipalities in their 2021 boundaries. We conduct extensive robustness checks to ensure the accuracy and reliability of the data we provide.

Our dataset has several key advantages. First, we provide a comprehensive database of election results for municipal, state, and federal elections at the municipal level since the 1990s. To our knowledge, no comparable database for municipal, state, and federal elections exists. Prior research – and the underlying datasets — often focus on select states or administrative levels higher than the municipality. Examples include mayoral elections in Bavaria between 1946 and 2009^3^, county-level election results for West Germany since 1953 (see https://github.com/cornelius-erfort/germany-53-21-districts), municipal-level election results in West Germany between 1949 and 1969^4^, and the partisanship of local mayors and district administrators based on annual reports of the Konrad Adenauer Foundation (Kommunales Wahllexikon) between 1990 and 2018^5^.

The closest dataset to ours was compiled by Rademacher (2018)^6^, who provides municipal council election data for many but not all states between 1990 and 2016 but does not address municipal boundary changes over time. Our dataset fills these gaps and provides the most comprehensive collection of municipal council election results between 1990 and 2021, together with state and federal election results.

Second, we provide harmonized data that addresses changes in administrative borders over time and challenges related to mail-in voting districts that cover multiple municipalities. Municipal reforms such as mergers present major obstacles in analyzing changes in voting patterns over time. We compile population-weighted crosswalk files such that historical electoral results are mapped onto 2021 administrative borders for a consistent panel of municipalities. Additionally, we provide shapefiles for administrative boundaries of municipalities and counties (districts) that allow researchers to easily match and aggregate election results to the desired level of analysis. We provide election results across three levels of government measured at the level of the municipality, allowing easy and reliable comparisons across space and over time. One limitation of our dataset is that it does not include elections to the Kreistag (county-level) or to the European Parliament because they are not consistently available at the level of the municipality over time.

Our dataset adds to recent efforts to collect data on local elections and local legislatures from several other contexts, such as the United States^7,8^ and Spain^9^. Our data enables progress on several research questions, including government responsiveness and representation, socio-economic drivers of partisan elections, and the role of municipal council partisanship on public service provision, government spending, and taxation. Since we provide harmonized data for three levels of government in Germany, scholars can further examine whether political decisions of one level of government have electoral consequences at other levels of government. Our data can be merged with municipal-level data on socio-economic covariates and spending patterns from INKAR (https://www.inkar.de/) or Wegweiser Kommune (https://www.wegweiser-kommune.de/), allowing research to study how socio-economic factors shape electoral behavior.

## Methods

This section details the construction and compilation process of our comprehensive German elections database, which includes municipal, state, and federal election results. The database is designed to provide a robust and reliable source of turnout and party electoral results at the level of the municipality for local, state, and federal elections. We systematically gathered raw election returns from various official sources, ensuring the inclusion of both major national parties and smaller local parties, where available. In addition, we propose solutions for various methodological challenges, such as discrepancies in reporting formats and electoral rules across states, the handling of mail-in voting districts, and boundary changes over time. Finally, given the increasing prevalence of ideologically extreme parties, we also provide aggregated vote shares of extremist parties.

### Municipal Elections

First, we compile data on municipal council elections (Stadtrat/Gemeinderat) from individual election result publications for every election-state combination. The Federal Statistical Office neither provides nor collects this data. We obtain most data as individual spreadsheets per election from each of the sixteen states’ election returning officers (Landeswahlleiter), either from their respective websites or via email. In several instances (Brandenburg 1993 and 1998; Hamburg 1991, 1993, and 1997; Rhineland-Palatinate 1994 and 1999; and Schleswig-Holstein 1994, 1998, and 2003), we obtained PDFs, which we digitized using the Optical Character Recognition (OCR) editor of ABBYY FineReader. Detailed data sources are listed in the README files online. In addition to manually checking the digitized data, we verified that the sum of raw votes for all municipalities (Gemeinden) matched the corresponding county (Landkreis) totals.

**Fig. 1:**
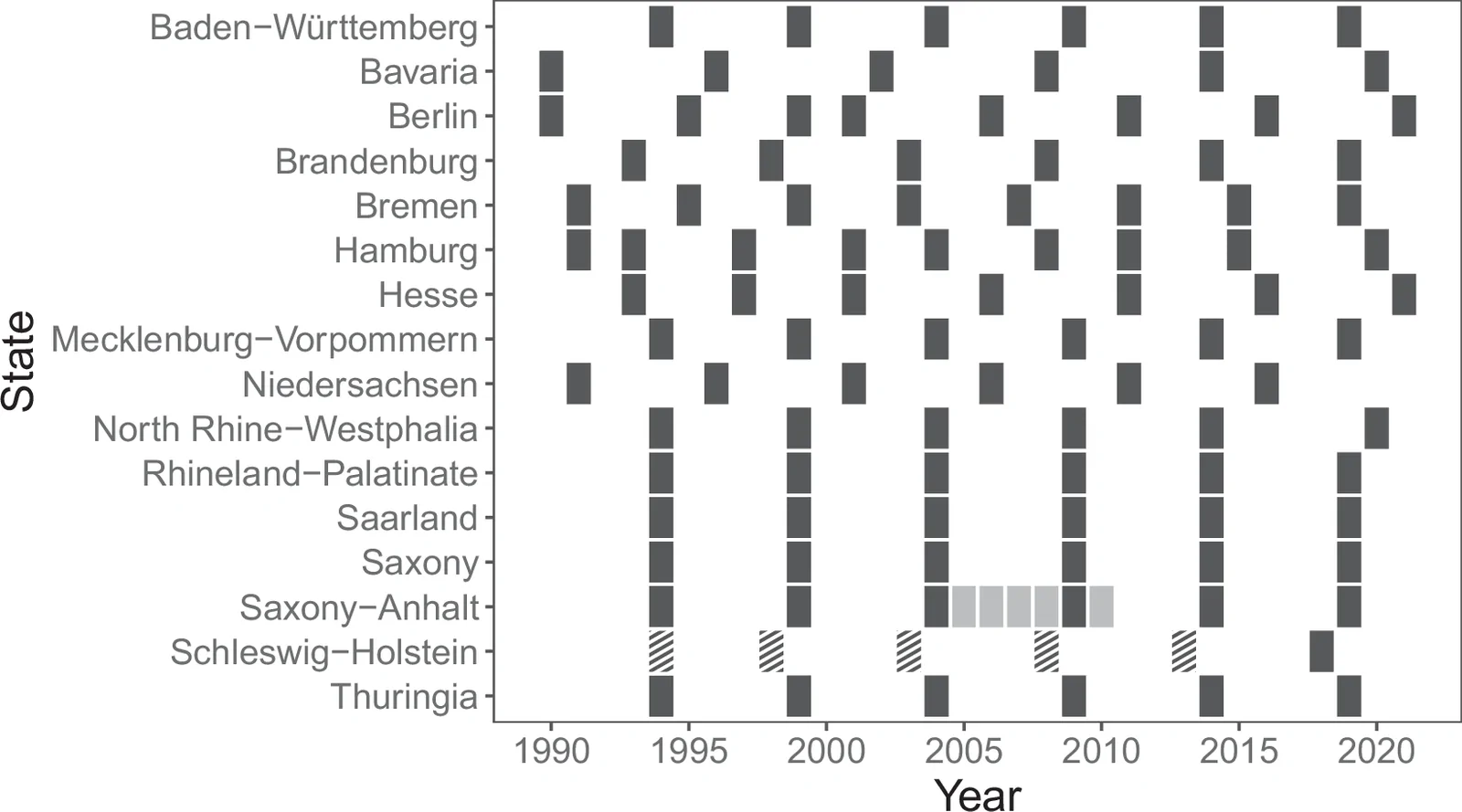
Municipal election data coverage, 1990–2020. *Notes:* Saxony-Anhalt had a municipal reform in 2007. Because of that some municipalities held elections before or after the official election date in 2009. Results for Schleswig-Holstein are only available for independent cities (‘kreisfreie Städte’) before 2018.

Figure 1 illustrates when municipal elections were held in each state between 1990 and 2020. We note that election data for Schleswig-Holstein before 2018 only include results for independent cities (‘kreisfreie Städte’). Data for municipalities that are part of a county are not available in any format for these elections, according to the state statistical office.

Not all states report election results in the same format, and even within a state, there are often over-time differences in how results are reported. As a result, there are several idiosyncrasies in the data that researchers should be aware of.

#### Differences in reported parties

Most importantly, all states report election results for the major national parties (SPD, CDU/CSU, FDP, Greens, Die Linke, and the AfD). In addition, states usually report results for Die PARTEI, Freie Wähler, and – for later elections – AfD and Piraten. We note that the data include full results for the AfD in all municipal elections held since its founding in 2013, with the exception of the 2014 election in Mecklenburg-Vorpommern, where official election reports only include results for the AfD in the state’s big cities (Rostock, Schwerin).

**Fig. 2:**
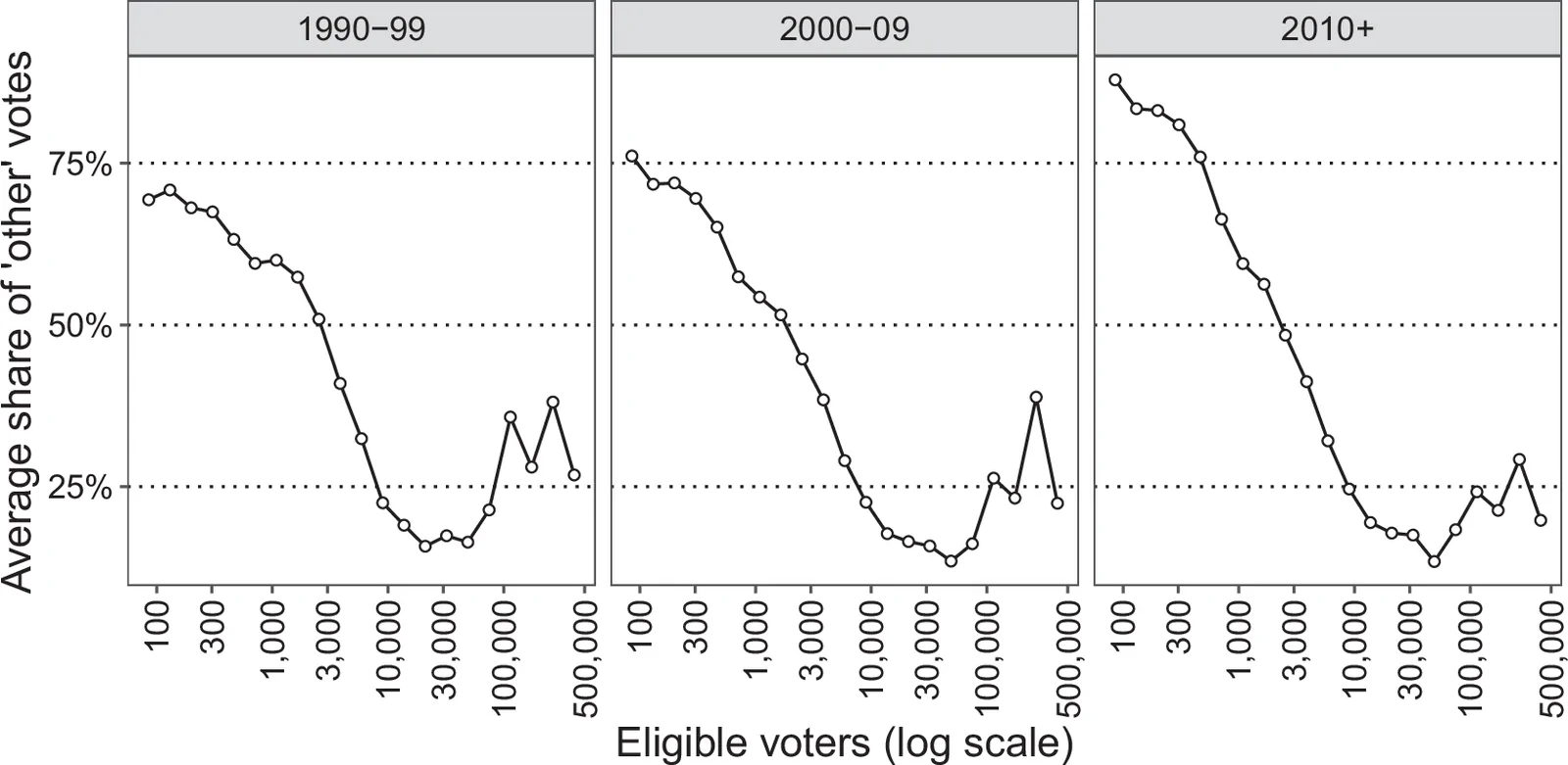
Average share of “Other” category vote shares in municipal elections as a function of number of eligible voters, by decade.

Small local parties and independent lists are not reported consistently: sometimes they are disaggregated at each locality, but sometimes they are reported as a separate “Other” category. To make election results as robustly comparable across time and states as possible, we hence report election results for all the major parties (SPD, CDU/CSU, FDP, Greens, Die Linke, and AfD) and, where available, for the Piraten, Die PARTEI, and Freie Wähler. We then calculate a remainder category (“Other”) by subtracting the sum of the votes for all parties for which disaggregated results are available from the total number of votes. In small municipalities, where often only a few candidates run for election and who are often not affiliated with any of the major parties, the “Other” category can amount to a substantial share of the overall votes. Hence, one has to be careful in comparing election results between larger and smaller municipalities. In Fig. 2, we present the share of the “Other” category as a function of eligible voters. Based on Fig. 2, our recommendation on this point is to check the robustness of any results to excluding municipalities with fewer than 3, 000 eligible voters.

Additionally, we note that the ‘Other’ category is inherently variable across elections and states, as it aggregates votes for small local parties, independent lists, and other entities that are often inconsistently reported. While it would be possible to disaggregate this category in some cases, it would come with significant challenges. First, election results for smaller municipalities often include numerous tiny parties and independent lists, which cannot be unambiguously assigned to major parties. Second, including all these minor lists in a harmonized dataset would make it both unwieldy – with potentially thousands of columns – and difficult to use for most comparative analyses. Finally, many election results provide only the major parties and an undifferentiated ‘Other’ category, making cross-time and cross-state comparisons impossible if more disaggregated data were included elsewhere. For researchers interested in exploring the detailed composition of the ‘Other’ category in a given state and municipal election, the original, unprocessed results files include the full disaggregation where available.

#### Differences in electoral rules

Additionally, electoral rules differ substantially across states on a number of dimensions, including the minimum age for candidates, whether lists are open or closed, and in terms of the total number of votes any one voter is allowed to cast. Table 1 provides an overview of the main features of municipal electoral systems by states (excluding the city-states Berlin and Hamburg) as of 2024. For example, North Rhine-Westphalia has closed-list (or mixed-member) proportional representation (PR) elections, with every voter allowed to cast one single vote. In contrast, Saxony holds open-list PR elections with every voter allowed to cast a total of three votes. Electoral systems with open lists allow voters to cast votes across lists (‘panaschieren’) or cast several votes within the same list (‘kumulieren’). The fact that in some states, any one voter may cast more than one vote leads to substantial differences in how votes are reported and translated into seats. Practically, this also means that the sum of absolute votes in our data often exceeds the number of voters, making the consistent interpretation of absolute votes difficult. Moreover, not all states report weighted election results. We therefore provide proportional vote shares as the most consistent and comparable results, as these are based on dividing the total raw votes cast for any one party by the total number of valid votes cast in a municipality.

**Table 1:** Overview of municipal electoral systems.

State	Period	Electoral system	Party list	Number of votes	Apportionment
Baden-Württemberg	5 years	open-list proportional	open	# of seats	Sainte-Laguë
Bavaria	6 years	open-list proportional	open	# of seats	Sainte-Laguë
Brandenburg	5 years	open-list proportional	open	3	Hare/Niemeyer
Bremen	4 years	open-list proportional	open	5	Sainte-Laguë
Hesse	5 years	open-list proportional	open	# of seats	Hare/Niemeyer
Mecklenburg-Vorpommern	5 years	open-list proportional	open	3	Hare/Niemeyer
Niedersachsen	5 years	open-list proportional	open	3	Hare/Niemeyer
North Rhine-Westphalia	5 years	mixed-member proportional	closed	1	Sainte-Laguë
Rhineland-Palatinate	5 years	open-list proportional	open	# of seats	Sainte-Laguë
Saarland	5 years	proportional	closed	1	d’Hondt
Saxony	5 years	open-list proportional	open	3	Sainte-Laguë
Saxony-Anhalt	5 years	open-list proportional	open	3	Hare/Niemeyer
Schleswig-Holstein	5 years	mixed-member proportional	closed	# of mandates	Sainte-Laguë
Thuringia	5 years	open-list proportional	open	3	Hare/Niemeyer

*Notes:* Electoral systems with open lists allow voters to cast votes across lists (“panaschieren”) or cast several votes within the same list (“kumulieren”). In Schleswig-Holstein, municipalities can have up to seven direct mandates per electoral district. *Sources:* https://www.wahlrecht.de/kommunal/, as of 2024.

### State Elections

**Fig. 3:**
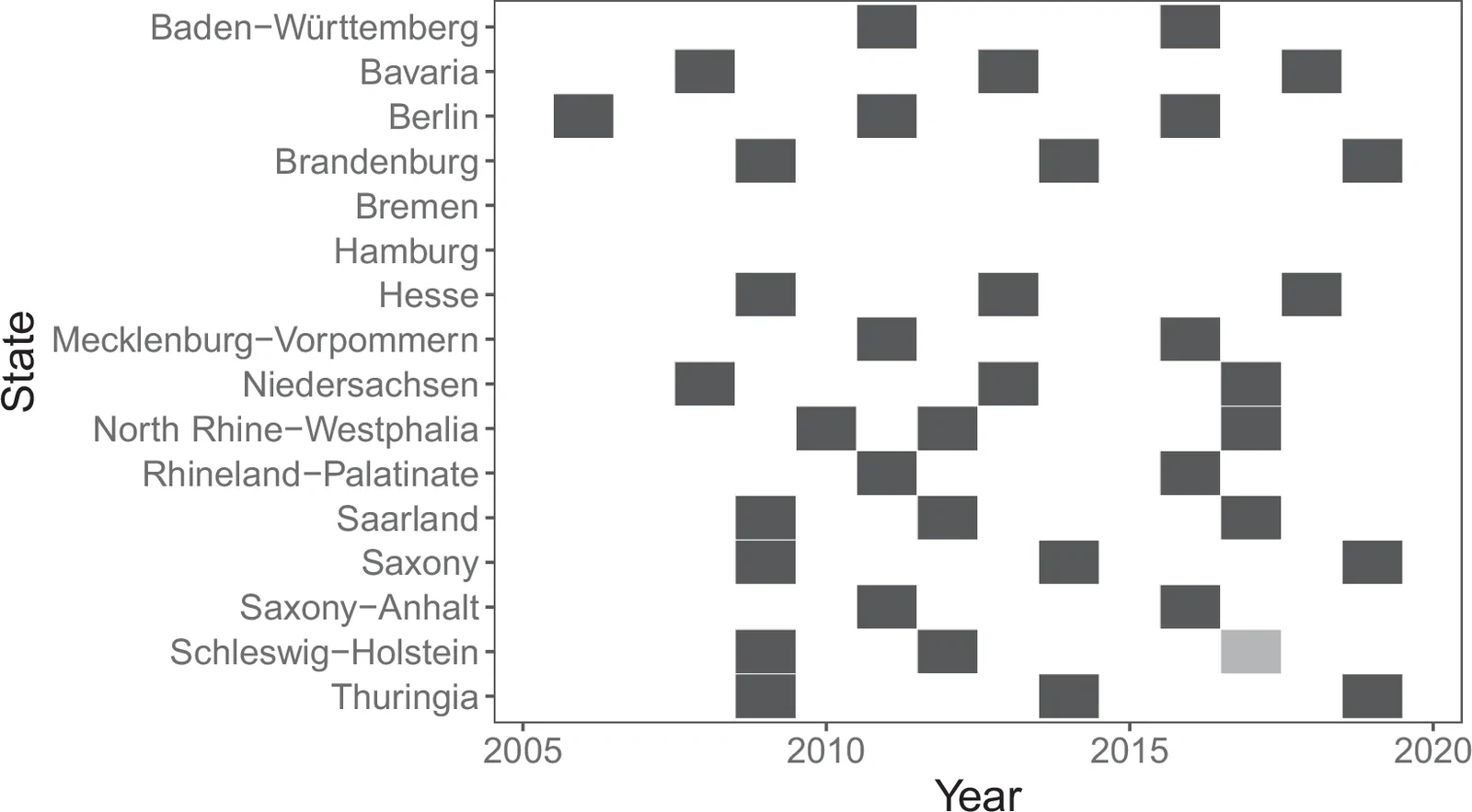
State election data coverage, 2006–2019. *Notes:* Official election data for Schleswig-Holstein from 2017 onward is unavailable at the municipal level^10^.

We collect official state election results at the municipal level from the regional data bank of the German Federal Statistical Office (DESTATIS)^10^. We retrieve and import the data using the wiesbaden R package^11^ and the SOAP XML web service of DESTATIS. This data is available for all elections in the 16 German states for the period 2006–2019. Note that official election data for Schleswig-Holstein from 2017 onward is unavailable at the municipal level due to the use of joint mail-in voting districts across multiple municipalities in some areas and the integration of mail-in votes with in-person voting districts in others, making consistent data representation impossible^10^. The data contains vote shares for CDU/CSU, SPD, FDP, Greens, and Die Linke, as well as for the AfD after 2012. We also provide a remainder category (“Other”) for the vote share of all other parties. Finally, we calculate the turnout and provide data on the total number of eligible voters and valid votes. Figure 3 illustrates when elections were held in each state during this period.

Note that the state elections data at the time of publication do not include mail-in voting data for Baden-Württemberg, Brandenburg, Mecklenburg-Vorpommern, Saxony, Saxony-Anhalt, and Thuringia.

### Federal Elections

Finally, we collect official federal election results from the Federal Returning Officer^12^. The German electoral law provides for a system of personalized proportional representation for federal elections. Each voter has two votes: the first for an individual constituency candidate, and the second for a party list in a particular state (*Zweitstimme*).

We collect all publicly available election results for the *secondary* vote at the municipality and county level. The municipality-level election results are available for all elections since 1980, while the county-level results have been released for all elections since 1953. We provide vote shares for all parties that ever ran in the time period covered by the respective data sets.

Some political parties in Germany have merged with others or changed their official names over time. For example, the present-day “Bündnis 90 / Die Grünen” (the Green party) is a combination of two parties: “Die Grünen,” which participated in all federal elections from 1980 to 2021, and “Bündnis 90,” which competed only in the 1990 federal election. To ensure consistency, we harmonize the vote shares for these parties across different election years, consolidating them under their current name. In addition to the harmonized data, we also provide the original vote shares for all parties, as recorded in each election, in the dataset federal_muni_raw. This allows researchers to analyze both the standardized data and the historical party configurations.

Further, we provide variables summarizing the vote shares of the following extremist parties: 1) far-right, 2) far-left (without Die Linke), and 3) far-left (with Die Linke). We provide two far-left variables because the party Die Linke had been under surveillance by the German Federal Office for the Protection of the Constitution (*Bundesamt für Verfassungsschutz*) for the period 2007–2014 because of “left-extremist aspirations”. These variables allow researchers to analyze pre-trends of extremist party families, even before specific parties of interest were established. For example, by calculating the vote share of far-right parties, one can assess pre-trends prior to the foundation of the AfD in April 2013, a party that did not receive votes before 2013 (see Heddesheimer, Hilbig, and Wiedemann (2024)^13^ or Heddesheimer, Hilbig, and Voeten (2024)^14^ for empirical applications of this procedure).

We gather information on extremist parties from reports from the German Federal Office for the Protection of the Constitution and compare them with the party family coding of the ParlGov project^15^. Table 2 lists all parties included in the three categories.

**Table 2:** Classification of Extremist Parties.

Category	Parties	Variable
**Far-right**	AfD (Alternative für Deutschland)	far_right
	BF_B (Bund Freier Bürger)	
	DDD (Bund der Deutschen Demokraten)	
	DG (Deutsche Gemeinschaft)	
	Die Rechte	
	DNS (Dachverband der Nationalen Sammlung)	
	DRP (Deutsche Reichspartei)	
	DSU (Deutsche Soziale Union)	
	DVU (Deutsche Volksunion)	
	FAP (Freiheitliche Deutsche Arbeiterpartei)	
	NPD (Nationaldemokratische Partei Deutschlands)	
	REP (Die Republikaner)	
	III. Weg	
**Far-left**	BSA (Bund Sozialistischer Arbeiter)	far_left
	BWK (Bund Westdeutscher Kommunisten)	
	DKP (Deutsche Kommunistische Partei)	
	KBW (Kommunistischer Bund Westdeutschland)	
	KPD (Kommunistische Partei Deutschlands)	
	SGP (Sozialistische Gleichheitspartei, formerly PSG)	
	SpAD (Spartakist-Arbeiterpartei Deutschlands)	
	V (Volksfront gegen Reaktion, Faschismus und Krieg)	
	Die Linke/PDS	far_left_wLinke

To facilitate linking GERDA with external party-level datasets, we provide a crosswalk file between all parties in our datasets and external identifiers from the ParlGov project. Specifically, the file named party_crosswalk.rds maps each party label in our dataset to the corresponding ParlGov identifier, which further allows merging with other databases such as the Comparative Manifesto Project^16^ or the European Election Study. The R package accompanying this paper, gerda, includes a party_crosswalk function to automatically map party names to their corresponding values from the ParlGov database.

### Comparability and Harmonization

We take several steps to provide comparable and harmonized data: 1) account for joint mail-in voting districts; 2) harmonize election data to 2021 municipal boundaries; and 3) account for changes in city-state electoral geography (Berlin, Hamburg, and Bremen).

#### Joint mail-in voting districts

The first challenge of municipality-level German election data is mail-in votes because some municipalities share mail-in voting districts. This affects only federal elections. Furthermore, since the 2009 federal election, some municipalities have their own unique mail-in voting district *and* an additional joint mail-in voting district. All municipalities in a county that form a joint mail-in voting committee receive the same digits at the end of their Official Municipality Key (*Amtlicher Gemeindeschlüssel* – AGS). The assignment of this number varies by election year and by whether all municipalities in that county share one singular joint mail-in voting committee or whether there are multiple mail-in voting committees in the respective county (a phenomenon that occurred, for example, in Berlin in the 2017 and 2021 elections). Information on this assignment procedure can be found in the “information appendix” (*btw_wbz_hinweise*) that the Federal Returning Officer issues.

**Fig. 4:**
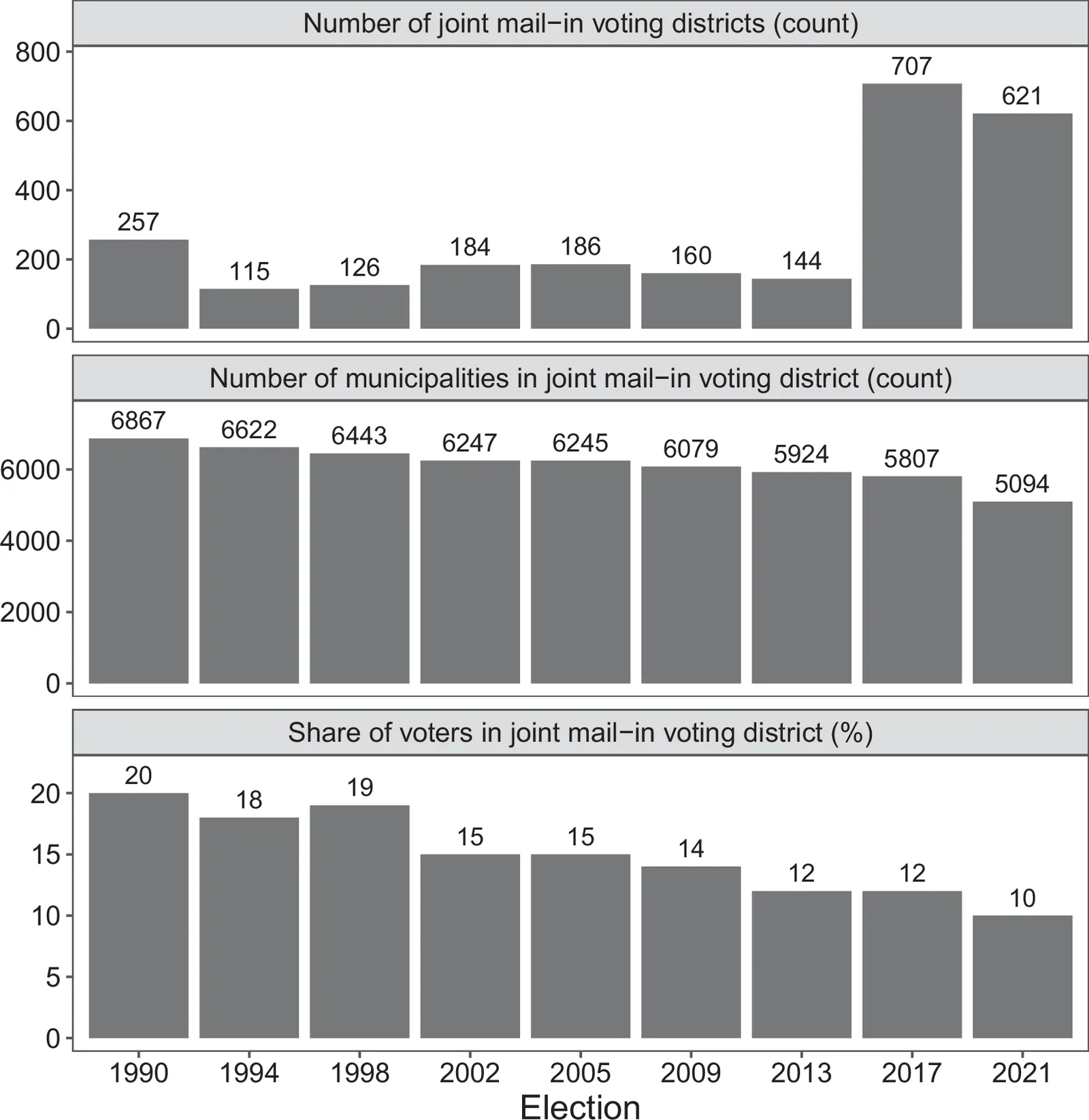
Significance of joint mail-in voting districts in each federal election. *Notes:* Statistics are based on the harmonized municipality-level federal election data.

Figure 4 presents the importance of joint mail-in voting districts for each federal election. While the number of distinct shared mail-in districts increased substantially in 2017, the number of municipalities covered by such districts decreased over time (from 64% of all municipalities in 1990 to 48% in 2021), as well as the number of eligible voters in these districts (from 20% to 10%).

To fully capture all votes, it is essential to consider the hierarchical structure that places municipalities within larger joint mail-in voting districts. As described above, we only observe in-person voting at the municipal level. It is not possible to unambiguously assign mail-in votes to a given municipality, since we only know the number of mail-in votes for the higher-level mail-in voting district, which comprises multiple municipalities. For researchers who prefer to neglect joint mail-in voting districts and focus on districts with unique mail-in votes, we make available a dataset (federal_muni_raw) that includes all municipalities with unique mail-in voting, as well as all additional joint mail-in voting districts. These additional “synthetic” districts can be identified by the last three digits of their Official Municipality Key (they end on ‘999’). We also added a variable to the dataset that takes the value ‘1’ if the row is a joint mail-in district (joint_mailin). Researchers who want to follow this approach should use this dataset and remove all observations identified as joint mail-in voting districts.

The significance of mail-in votes is underscored by their increasing prevalence; mail-in votes rose from 24.3% in the 2013 federal election to 28.6% in 2017 and 47.3% in 2021^12^. Neglecting mail-in votes in the analysis and focusing only on in-person votes therefore omits a large share of cast votes.

We propose and implement an approach to address these challenges and maintain consistency in units throughout elections. In particular, we suggest distributing joint mail-in votes across municipalities that belong to the same joint mail-in voting district, using the number of municipal-level voters with a polling card (*Wahlschein*), which serves as a notification and proof of voter eligibility, as weights. In German federal elections, voters have to apply for a polling card if they want to vote by mail or at a different polling station in their constituency. The number of people with a polling card in each municipality is recorded in all raw federal election files.

Consider a municipality *i* that belongs to a joint mail-in voting district *d*. Let ${Y}_{i}^{\,{\rm{ip}}}$ denote the *in-person* votes for some party in municipality *i*, and let ${Y}_{d}^{\,{\rm{mail}}}={\sum }_{i\in d}{Y}_{i}^{mail}$ be the *total mail-in* votes for the entire district *d*. Define *w*_*i*_ as the number of polling card voters (i.e., individuals eligible to vote by mail or outside their home polling station) in municipality *i*. The total polling card voters in district *d* is

$${w}_{d}=\sum _{i\in d}{w}_{i}.$$

The *combined* votes for municipality *i*, denoted ${\widetilde{Y}}_{i}^{\,{\rm{total}}}$, are then given by

$${\widetilde{Y}}_{i}^{{\rm{total}}}={Y}_{i}^{{\rm{ip}}}+\frac{{w}_{i}}{{w}_{d}}\,{Y}_{d}^{{\rm{mail}}},$$

where the fraction $\frac{{w}_{i}}{{w}_{d}}$ serves as a polling-card-based weight for allocating the district-level mail-in votes ${Y}_{d}^{{\rm{mail}}}$ back to municipality *i*. This weighting approach reflects the idea that the number of polling card voters (*w*_*i*_) is a good proxy for the number of mail-in ballots likely originating in municipality *i*. We provide a dataset (federal_muni_unharm) that implements this procedure for all German municipalities.

This approach has two advantages: (i) we do not have to discard mail-in votes, and (ii) we can keep all municipalities in the dataset. However, this approach rests on the assumption that mail-in votes – that is, both the total number and the number of votes for particular parties – can be homogeneously distributed to each municipality based on the number of polling card voters. This means that the proportion of mail-in votes for each party in a joint mail-in voting district is expected to be consistent across all municipalities within that district. This is a strong assumption, which is almost certainly violated in most cases. Since it is impossible to reconstruct the actual number of mail-in votes for each party per municipality, we consider our approach to be an approximation.

An alternative would be to aggregate units to the joint mail-in district level. This approach creates synthetic units. Joint mail-in districts are treated as a single entity for analysis purposes, combining all municipalities within the district into one unit (see Ferwerda and Riaz (2024)^17^ for an application of this approach). The advantage of this approach is that it ensures full capture and accurate representation of all mail-in votes within the district. However, in doing so, this approach also leads to a loss of granularity. This is particularly unfavorable in settings where the “treatment” is administered at the municipality level. We have chosen not to implement this approach. Researchers can use the raw data set we provide (federal_muni_raw) and create these synthetic municipalities by summing the voting variables of all municipalities that share the same joint mail-in voting district.

One complication in our approach arises when calculating voter turnout in municipalities that are part of joint mail-in voting districts. In particular, we identified 90 municipality-year observations, across nine different states, where the number of total votes received exceeded the number of eligible voters in municipalities after adjusting for mail-in votes. To ensure that turnout values remain within a plausible range in these instances, we recalculated turnout by excluding mail-in votes, considering only the ratio of in-person votes to eligible voters. These cases are flagged in both the unharmonized and harmonized datasets using the variable flag_naive_turnout_above_1. We also provide an additional variable, turnout_wo_mailin, which calculates the turnout as the ratio of total in-person votes to eligible voters for all municipalities and elections.

#### Examining measurement error due to joint mail-in voting districts

To evaluate the measurement error introduced by our mail-in vote distribution procedure, we construct distributed mail-in districts and compare municipalities’ total votes (in-person plus mail-in) under our distribution approach versus their observed total votes. Let ${\mathcal{I}}$ be the set of municipalities that do not share a mail-in district (i.e., each municipality $i\in {\mathcal{I}}$ has its own unique mail-in vote count). There are 5,552 municipalities in this set. Denote by *w*_*i*_ the number of polling card voters in municipality *i*, let ${Y}_{i}^{{\rm{ip}}}$ be its in-person votes, and ${Y}_{i}^{{\rm{mail}}}$ its observed mail-in votes. Hence, the observed total votes are

$${Y}_{i}^{{\rm{total}}}={Y}_{i}^{{\rm{ip}}}+{Y}_{i}^{{\rm{mail}}}.$$

We proceed as follows:

1. **Subset the data:** Restrict the dataset to all municipalities $i\in {\mathcal{I}}$. For each of these, we observe ${Y}_{i}^{{\rm{ip}}}$, ${Y}_{i}^{{\rm{mail}}}$, and *w*_*i*_.
2. **Create 1,000 distributed mail-in districts (k-means):** We use the centroid coordinates of municipalities to create spatially contiguous districts via a *k*-means algorithm, aiming for 1, 000 districts total. This choice approximates the real average district size in terms of the number of municipalities per district.
3. **Aggregate mail-in votes and redistribute:** For each distributed district *d* ∈ {1, …, 1, 000}, let
  $${Y}_{d}^{{\rm{mail}}}={\sum }_{i\in d}{Y}_{i}^{{\rm{mail}}}$$
  be the total distributed mail-in votes. We then redistribute ${Y}_{d}^{\,{\rm{mail}}}$ back to each municipality *i* ∈ *d* based on polling card voters:
  $${\widetilde{Y}}_{i}^{\,{\rm{mail}}}=\frac{{w}_{i}}{{\sum }_{j\in d}{w}_{j}}\,{Y}_{d}^{\,{\rm{mail}}},$$
  mirroring our procedure described previously. Next, we form a distributed total vote count for *i*,
  $${\widetilde{Y}}_{i}^{{\rm{total}}}={Y}_{i}^{{\rm{ip}}}+{\widetilde{Y}}_{i}^{{\rm{mail}}},$$
  which combines in-person votes and redistributed mail-in votes.
4. **Compare actual vs. distributed vote shares:** For each party *p*, let ${s}_{i}^{p}=\frac{{Y}_{i}^{p}}{{Y}_{i}^{\,{\rm{total}}}}$ and ${\widetilde{s}}_{i}^{p}=\frac{{\widetilde{Y}}_{i}^{p}}{{\widetilde{Y}}_{i}^{\,{\rm{total}}}}$, where ${Y}_{i}^{p}$ and ${\widetilde{Y}}_{i}^{p}$ are the observed and redistributed votes for party *p*. Then define the municipality-level difference in vote shares as
  $${\Delta }_{i}^{p}={\widetilde{s}}_{i}^{p}-{s}_{i}^{p},$$
  and also consider its absolute value $| {\Delta }_{i}^{p}|$. These measures capture the measurement error introduced by our polling-card-based allocation method in terms of vote-share differences rather than raw vote counts.

We conduct this procedure for the 2021 election results using *D* = 1, 000 distributed k-means districts. For each municipality *i* and each of the six major parties, we calculate both ${\Delta }_{i}^{p}$ and $| {\Delta }_{i}^{p}|$. We report two kinds of means for each party: *unweighted* (an unweighted average across municipalities) and *weighted* (using each municipality’s total distributed votes as weights). In the weighted case, the overall mean of ${\Delta }_{i}^{p}$ across all municipalities is mechanically zero, since any over-allocation of shares in some municipalities is offset by under-allocations in others within the same district.

**Fig. 5:**
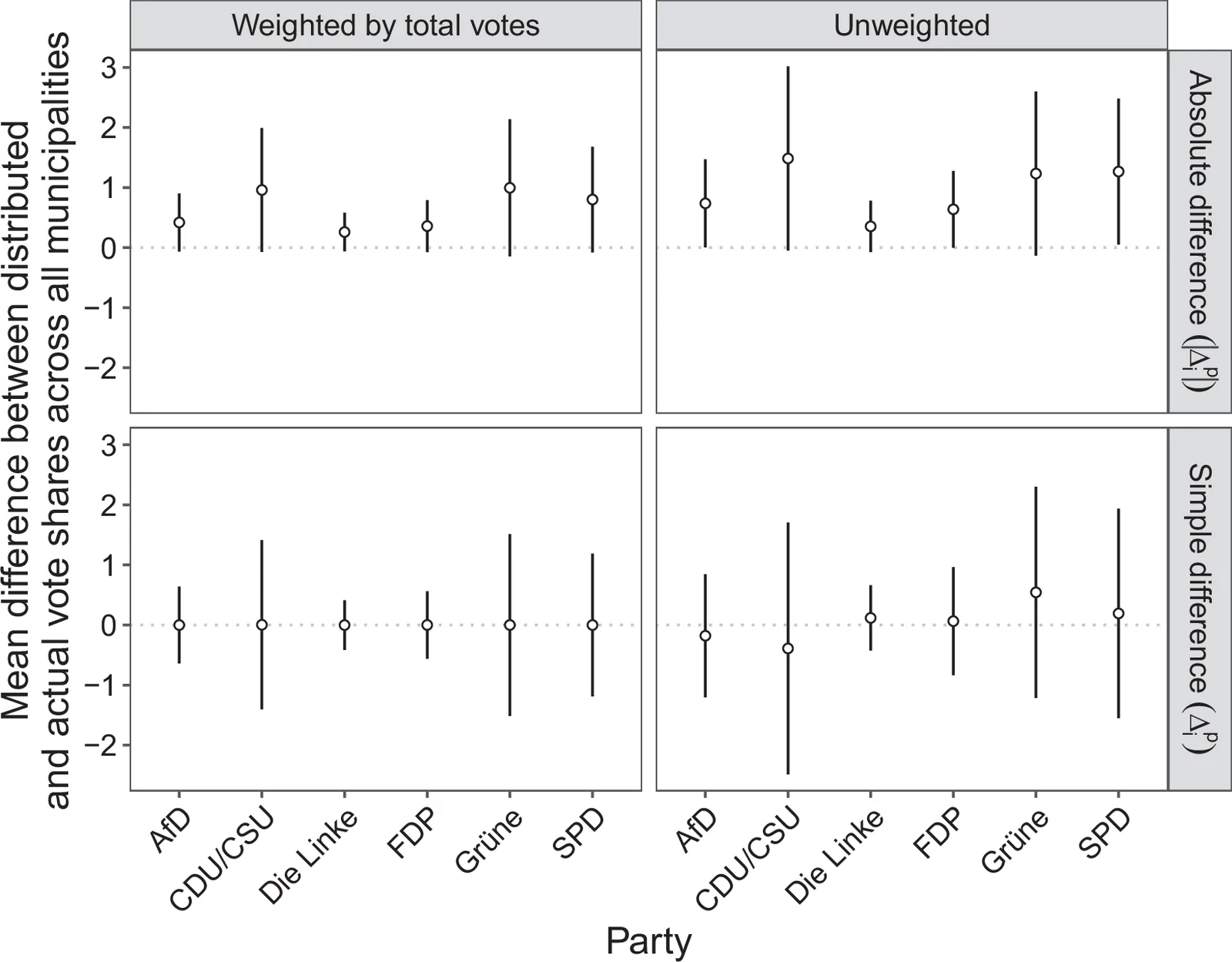
Measurement error due to distributing mail-in votes in 2021 (based on 1, 000 distributed k-means districts). *Notes:* We report the mean of ${\Delta }_{i}^{p}$ and the mean of $| {\Delta }_{i}^{p}|$ (in percentage points) by party, both unweighted and weighted by total distributed voters (i.e. weights are ${\widetilde{Y}}_{i}^{{\rm{total}}}$). The difference is defined as distributed vote share minus actual vote share for a given party. Vertical bars indicate ±1 standard deviation (SD). Note that the vertical bars can include values smaller than zero for the absolute difference since we present SDs rather than, for example, the percentile of the distribution of $| {\Delta }_{i}^{p}|$. However, the absolute difference itself is never below zero. For the weighted sample, standard deviations shown in this plot are also computed using the total distributed votes ${\widetilde{Y}}_{i}^{{\rm{total}}}$ as weights.

Figure 5 reports the mean difference in vote shares, ${\Delta }_{i}^{p}$, and the mean absolute difference, $| {\Delta }_{i}^{p}|$, for these six parties, in both unweighted and weighted forms. As expected, the weighted average difference in the bottom left panel is always 0. When examining absolute differences, which measure the distance between distributed and actual vote shares, we find somewhat larger deviations for the CDU/CSU, Greens, and SPD than for the other parties. However, these deviations, on average, do not exceed 1.5 percentage points in the unweighted sample or 1 percentage point in the weighted sample. Because weighting by total distributed voters mitigates some of the measurement error introduced by mail-in vote redistribution, researchers using our data may consider showing both weighted and unweighted results.

#### Harmonization to 2021 boundaries

A second challenge of municipality-level German election data comes from boundary changes of municipalities and counties over time. For example, in 2023, there were 43 municipality changes: 18 dissolutions, 10 partial separations, 10 municipality key changes, and 5 municipality name changes, according to the German Federal Statistical Office. These changes complicate the comparison of election results across different time periods because the geographic units being compared are not consistent. This inconsistency can lead to inaccuracies if the data is not properly adjusted for boundary changes.

**Fig. 6:**
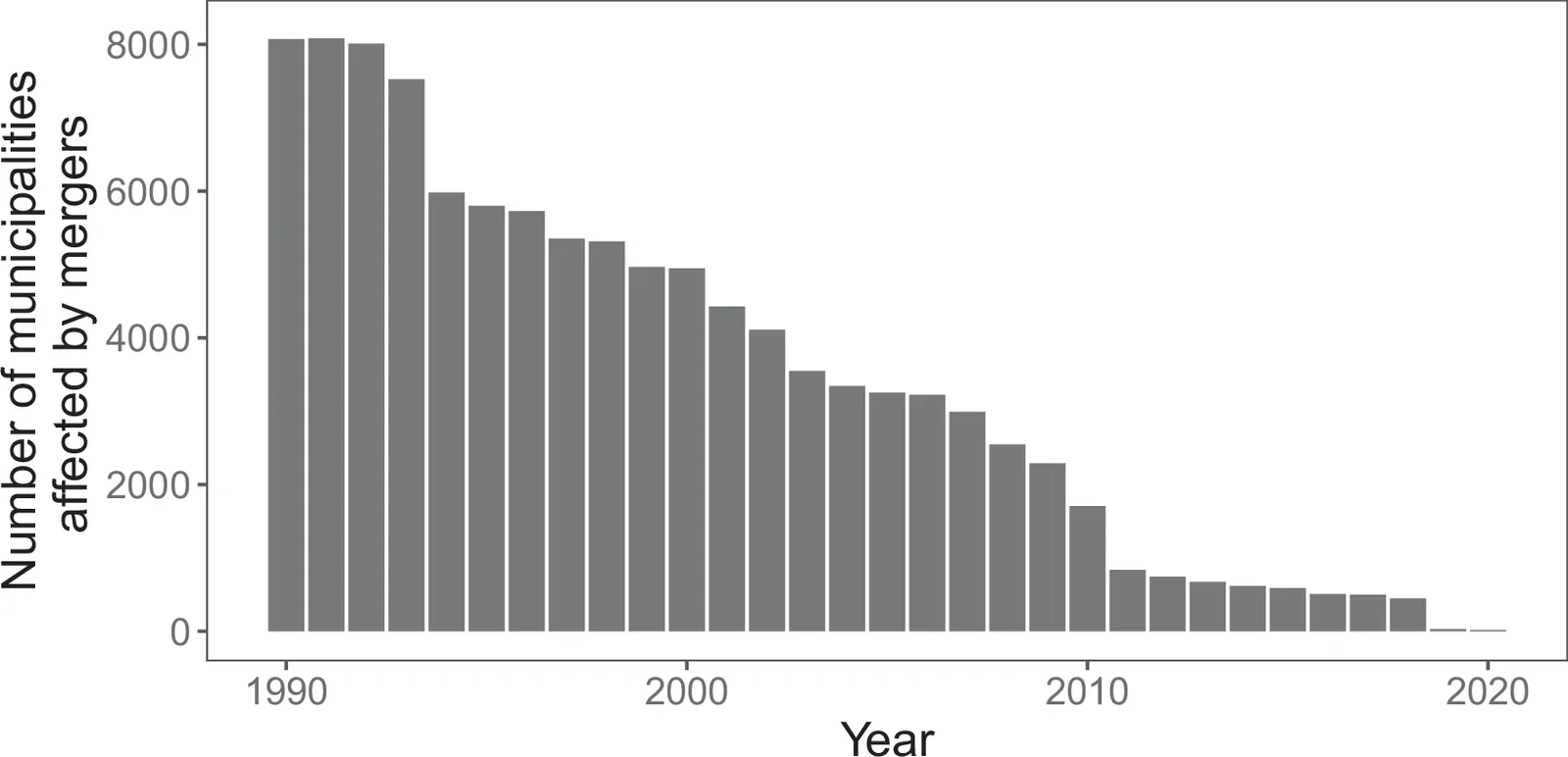
Number of municipalities affected by boundary changes and/or mergers per year. *Notes:* A municipality is affected by a boundary change, merger, or partition if its AGS key is different from its 2021 AGS key or if its population-weighted crosswalks are smaller than 1.

Figure 6 illustrates the prevalence of these boundary changes.

To create balanced panels for all election datasets – municipal, state, and federal – where the number of municipalities or counties remains constant over time, we map the boundary of each municipality or county in the years prior to 2021 to its 2021 boundaries using crosswalk files from the Federal Institute for Research on Building, Urban Affairs, and Spatial Development^18,19^. Specifically, for all municipalities or counties that were subject to border changes and/or mergers, we calculate a weighted average of each vote variable using a population-weighted key. For example, if municipalities *i* and *j* merge into a new municipality *k*, the value of a harmonized variable ${\widetilde{X}}_{k}^{{\rm{harm}}}$ (e.g., turnout) for municipality *k* after the merge is the population-weighted average of the values *X*_*i*_ and *X*_*j*_. That is,

$${\widetilde{X}}_{k}^{\,{\rm{harm}}}=\frac{{p}_{i}}{{p}_{i}+{p}_{j}}{X}_{i}+\frac{{p}_{j}}{{p}_{i}+{p}_{j}}{X}_{j},$$

where *p*_*i*_ and *p*_*j*_ are the population sizes of municipalities *i* and *j* on December 31 of the respective year. We create harmonized election datasets for all three types of elections (municipal, state, and federal) and denote them with the suffix “_harm”.

During the harmonization process, we encountered several inconsistencies in the official AGS identifiers across various datasets. As detailed in section “Incongruent municipality keys,” we systematically adjusted AGS keys, corrected inconsistencies in crosswalk years, and manually updated missing or inaccurate data to ensure accurate harmonization. Furthermore, in the context of federal elections, our approach of distributing joint mail-in voting district data by population weights introduced minor discrepancies in the calculation of total valid votes. Similarly, the harmonization of state election data results in a few cases for which the sum of all vote shares exceeds 1. We discuss these issues in section “Incongruencies in total votes.”

#### City states

A third challenge of German electoral data relates to city-states – cities that are simultaneously classified as states – and the changing granularity of election data provided by the Returning Officer. Germany has three city-states: Berlin, Bremen, and Hamburg. In the available municipal-level election data, Berlin was considered a single district in 1990, but since 1994, voting data were divided into several districts with distinct municipality keys. Similarly, Hamburg functioned as one district between 1990 and 2017 but was split into multiple districts in 2021. The city-state of Bremen has been consistently comprised of two voting districts (Bremen and Bremerhaven). In both the unharmonized and harmonized data, we aggregate all electoral data for Berlin and Hamburg to a single district level, respectively, across all years. For Bremen, we report voting results for both voting districts. However, in the raw unharmonized data, we do not adjust the districts and provide data on all original districts.

## Data Records

We make all data available through the German Election Repository and Data Archive (GERDA) at http://www.german-elections.com/and at the Harvard Dataverse at https://doi.org/10.7910/DVN/MD1E0J^20^. The repository contains unharmonized and harmonized datasets for federal, state, and municipal elections. The harmonized datasets provide consistent panels of municipalities in their 2021 boundaries. All datasets are available in .csv and .rds formats. The repository also hosts the crosswalk files for harmonizing the data, shapefiles for municipality and county boundaries in 2000 and 2021^21^, and area, employee, and population estimates based on the crosswalk files and harmonized to 2021 boundaries. Raw source files for federal and municipal elections are available on GitHub at https://github.com/awiedem/german_election_data/tree/main/data in folders designated raw in subdirectories /federal_elections and /municipal_elections, respectively. Table 3 lists the complete set of datasets.

**Table 3:** Datasets.

Data	Geographic level	Time period	Harmonization?	Name
Municipal elections	Municipality	1990-2020	No	municipal_unharm
Municipal elections	Municipality	1990-2020	Yes	municipal_harm
State elections	Municipality	2006–2019	No	state_unharm
State elections	Municipality	2006–2019	Yes	state_harm
Federal elections	Municipality	1980–2021	No	federal_muni_raw
Federal elections	Municipality	1980–2021	No	federal_muni_unharm
Federal elections	Municipality	1990–2021	Yes	federal_muni_harm
Federal elections	County	1953–2021	No	federal_cty_unharm
Federal elections	County	1990–2021	Yes	federal_cty_harm
Crosswalks	Municipality	1990–2021	—	ags_crosswalks
Crosswalks	County	1990–2021	—	cty_crosswalks
Shapefiles	Municipality	2000, 2021	—	VG250_GEM
Shapefiles	County	2000, 2021	—	VG250_KRS
Crosswalk covariates	Municipality	1990–2021	Yes	ags_area_pop_emp
Crosswalk covariates	County	1990–2021	Yes	cty_area_pop_emp

*Note:* File Structure of the GERDA Repository (http://www.german-elections.com/election-data/).

We plan to continuously update the data on the GERDA website to include new elections as results become available, and to incorporate any future boundary changes into the existing and newly released data.

In addition to downloading the data files from the GERDA website, researchers can also use the R package gerda to load the data directly into R. The package is available at https://cran.r-project.org/web/packages/gerda/index.html.

## Technical Validation

For all datasets, we perform several “sanity checks” to assess whether the values of the electoral variables are as expected and to check for irregularities in the harmonized data. Such irregularities may arise from rounding when aggregating vote shares due to municipal mergers, or from the aforementioned procedure to distribute joint mail-in votes across municipalities.

First, we confirm that party vote shares for a given unit–election combination sum to 1. Below, we discuss how we dealt with a few cases in which total party vote shares exceeded 1 as a result of the harmonization to 2021 boundaries. Second, we make sure that all party vote shares are bounded by 0 and 1. Third, we assess changes in (i) valid votes, (ii) party vote shares, and (iii) turnout between consecutive elections within a given county or municipality. We do this to detect possible data irregularities that would show up as very large percentage changes in the number of valid votes, party vote shares, or turnout between elections. We mostly did not encounter irregular patterns or reasons for concern across these three checks. However, we found several discrepancies regarding incongruent municipality keys, as well as turnout and total votes calculations. We describe how we addressed these incongruencies in the following. Fourth, we aggregate our harmonized municipal-level dataset of federal election results to the state-year-party level and compare the results to the election results that the Federal Statistical Office releases on their website.

### Incongruent municipality keys

We identified several cases in the original data where the AGS keys did not match any official crosswalk files: 1,056 municipal election observations and 86 state election observations, as well as 1,657 cases of municipal- and 67 cases of county-level results for federal elections. This discrepancy suggests that these AGS identifiers were incorrectly assigned or that the original data for these municipalities had been recorded for a municipality that did not exist at that point (i.e., had been merged into a different municipality, for example). Column flag_unsuccessful_naive_merge in the harmonized datasets flags the cases where we had to adjust the AGS.

To address this issue, we manually matched inconsistent AGS identifiers in the unharmonized, original election data with their correct counterparts from crosswalk files by referencing election result sources and information from official crosswalk files on year-specific AGS key changes. For each incorrect AGS key, we identified its correct match based on the election year and AGS key. We further implemented year adjustments – where necessary – to ensure that the AGS keys corresponded to the correct time period, particularly for municipalities or counties affected by boundary changes or mergers.

### Incongruencies in total votes

#### Federal elections

**Fig. 7:**
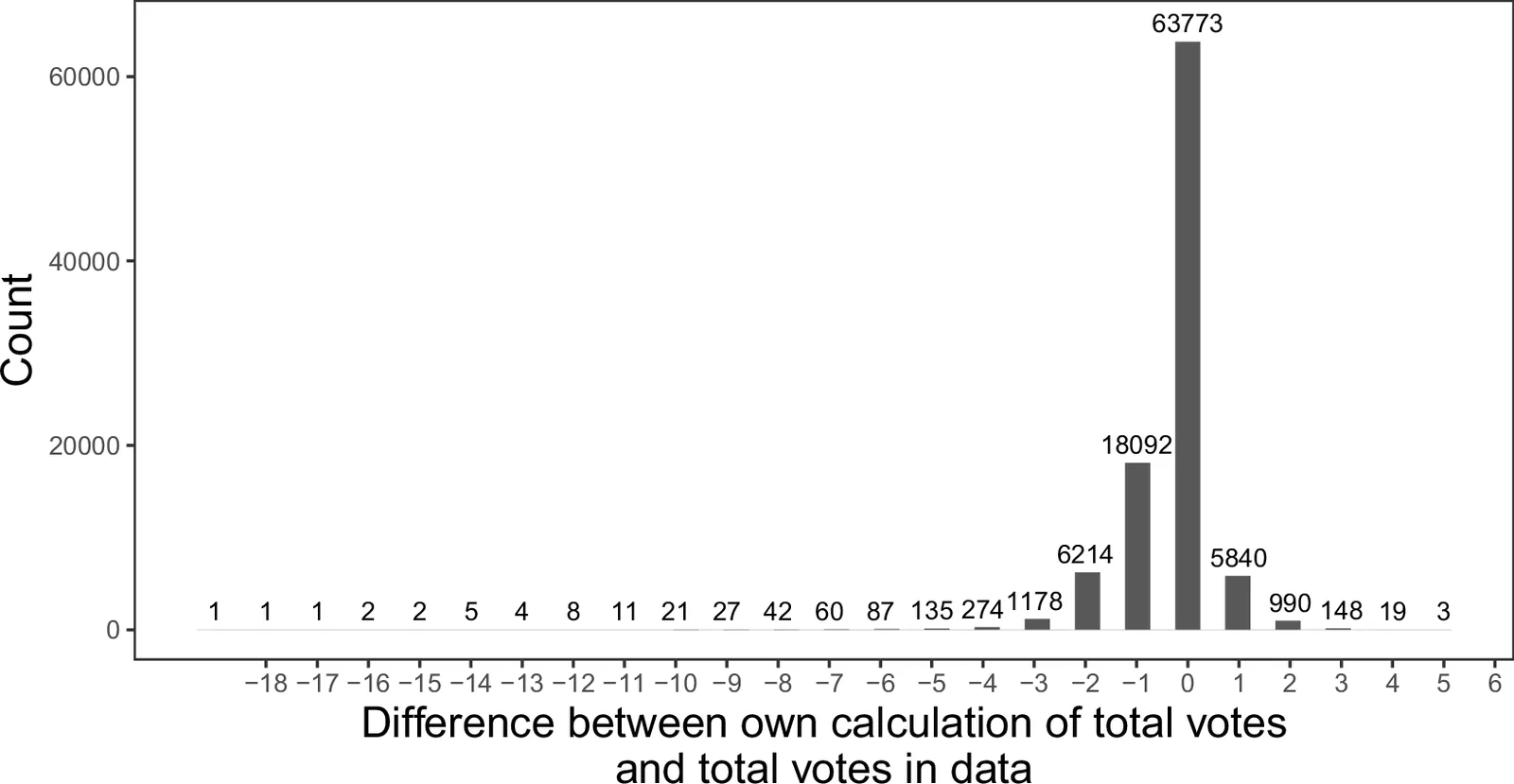
Incongruence between own calculation of total votes after accounting for joint mail-in districts vs. official total votes.

For federal elections, the boundary cross-walking procedure combined with our preferred approach of dealing with joint mail-in voting districts, that is, distributing such districts by population weights, leads to another challenge with respect to the total valid votes counted. Specifically, in some cases, the total number of votes calculated by summing up all votes for all parties in a municipality for an election differs from the measure of valid votes provided by the Federal Officer for that municipality. This issue arises from rounding the votes when distributing joint mail-in district votes by population weights to the municipalities. Because we provide vote *shares* in the harmonized data set, we have to use the number of total valid votes cast as the denominator. We choose the total number of votes that we calculate as the sum of all votes cast *after* votes are distributed from the joint mail-in voting districts. Note that we deem this choice to be not very influential. Figure 7 shows that there are no incongruencies between the official measure of valid votes and our own calculation of valid votes in the majority of cases (66%). Furthermore, the maximum difference between the two measures is 18 votes and, in most cases, the difference does not exceed two votes. We added a variable in the harmonized dataset that flags cases where the two measures are incongruent (flag_total_votes_incongruent).

#### State elections

Similarly, the harmonization of party vote shares in the state election data results in 29 observations for which the sum of vote shares exceeds 1, although the maximum deviance from 1 is 0.02. We add a variable that flags these cases (flag_total_votes_incongruent).

### Validation of aggregated harmonized federal election data against official state-level results

As a final check, we calculate whether aggregating our harmonized municipal-level dataset of federal election results to the state-year-party level matches the election results that the Federal Statistical Office releases on its website. We do this for three elections for which the Federal Statistical Office releases state-level results – 2013, 2017, and 2021. For each state-year-party cell, we calculate the number of votes based on the harmonized municipal-level data. We then calculate the absolute difference and relative deviation (the absolute value of the difference, divided by the total count in the official data) between the state-year-party cells in our own data and the official state-year-party data released by the Federal Statistical Office. For 41% of all 281 state-year-party cells, the number of votes in our dataset and the number of votes in the official data is exactly the same. For an additional 33% of all cells, the relative deviation between the two data sets is below 0.01%. For the remaining 26% of all cells, deviations range from 0.021% at the 80^th^ percentile and 0.035% at the 90^th^ percentile to 0.077% at the maximum of the distribution. Since the deviation between our data and the official data is never larger than 0.1% (for 90% of cells it is below 0.035%), we are confident that our data accurately reflect the correct number of votes.

## Usage Notes

**Fig. 8:**
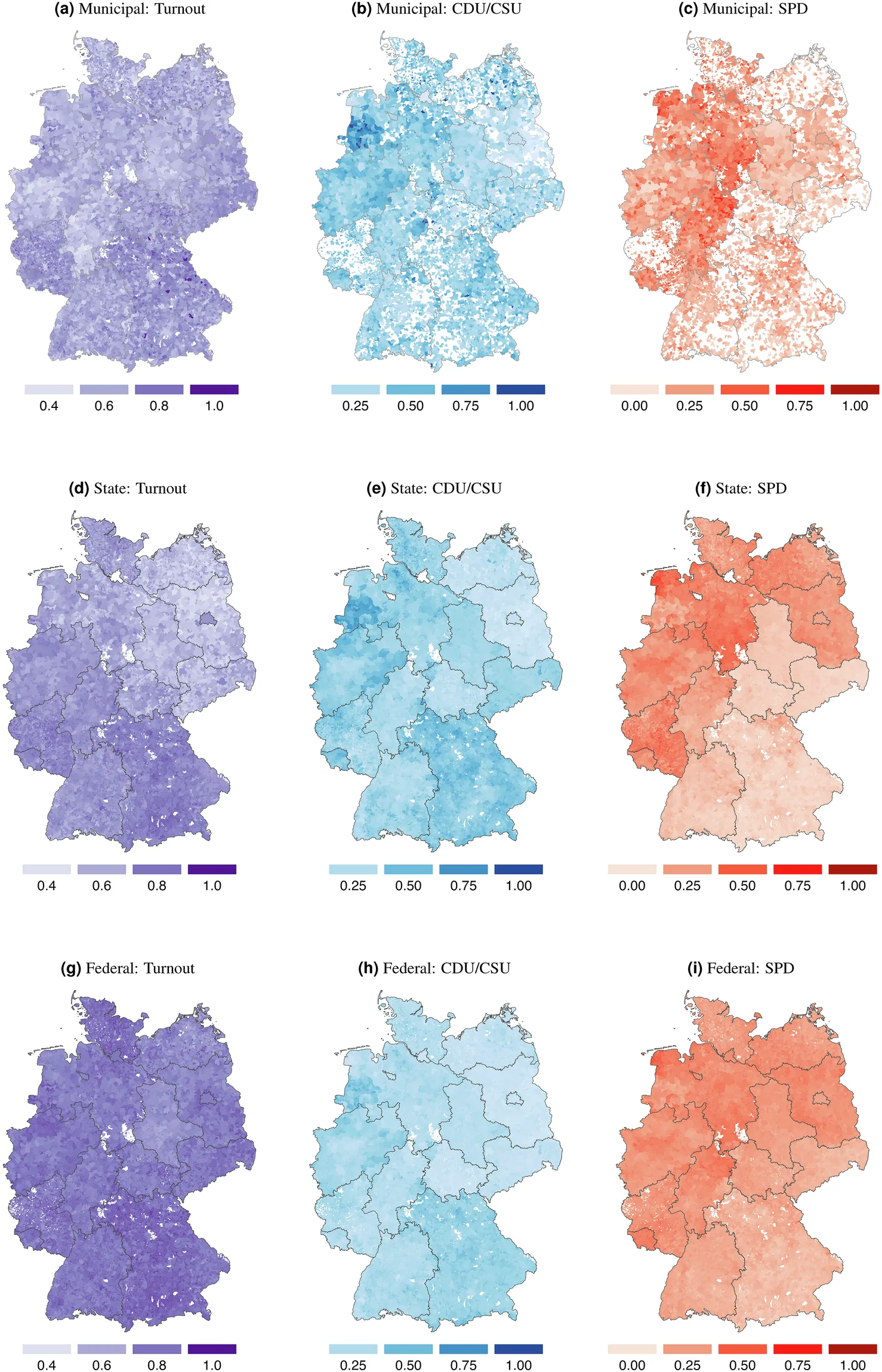
Maps of Municipal-Level Turnout and Party Vote Shares for Municipal, State, and Federal Elections. *Notes*: Data for 2021 federal elections and most recent state and municipal elections.

Figure 8 shows maps of turnout and vote shares for the two historically dominant parties in Germany, the Christian Democrats and the Social Democrats, in the most recent municipal and state elections as well as the 2021 federal election. All maps display considerable variation in turnout and party vote shares across and within state boundaries. As discussed above, in some states there is a large share of municipalities in which the vote shares of major parties are zero. These differences arise not due to data missingness but because of differences in reporting standards and electoral rules across states. While some states report candidates of major parties and aggregate party vote shares, others do not and, instead, only report results for lists or individuals that are not attributable to any of the major parties. This is often the case in very small municipalities where individual candidates run for office without party labels. For federal and state elections, we have election results for all municipalities. The remaining white areas in the maps in Fig. 8 are either lakes or forested areas ("gemeindefreies Gebiet”) or belong to the “Other” party category in municipal elections (Fig. 8 panels b and c).

## Data Availability

We digitized PDFs of local election data using the OCR editor of ABBYY FineReader. All other data processing and analysis were conducted using RStudio version 2024.12.0+467. The datasets and the code to replicate the main and supplementary analyses are publicly available at https://doi.org/10.7910/DVN/MD1E0J. More information, including harmonized and unharmonized data, boundary shapefiles, codebook, and a detailed explanation of the data, is available on our companion website at http://www.german-elections.com/.
