# Author Correction: GERDA: The German Election Database

**DOI:** 10.1038/s41597-025-06139-6

**Published:** 2025-10-23

**Authors:** Vincent Heddesheimer, Hanno Hilbig, Florian Sichart, Andreas Wiedemann

**Affiliations:** 1 Princeton University, Department of Politics, Princeton, NJ 08544 USA; 2 University of california, Davis, Department of Political Science, Davis, CA 95616 USA

Correction to: *Scientific Data* 10.1038/s41597-025-04811-5, published online 14 April 2025

In this article the caption for Table 2 incorrectly read, ‘Aggregated Vote Shares of Extremist Parties’ and should have read ‘Classification of Extremist Parties’. The original article has been corrected.

